# Decreased Endothelial Progenitor Cells Are Associated with Severe Coronary Artery Disease: Insights from a Clinical Study

**DOI:** 10.3390/jcdd12040132

**Published:** 2025-04-03

**Authors:** Ivan Tomić, Ivan Zeljko, Ivica Brizić, Violeta Šoljić, Ivona Ivančić, Monika Tomić, Marina Ćurlin, Domagoj Tomić

**Affiliations:** 1 Department of Internal Medicine, University Clinical Hospital Mostar, Kralja Tvrtka bb, 88000 Mostar, Bosnia and Herzegovina; ivan.zeljko9@gmail.com (I.Z.); ibrizic@gmail.com (I.B.); monika.tomic@gmail.com (M.T.); 2 Faculty of Health Study, University of Mostar, Trg Hrvatskih Velikana 1, 88000 Mostar, Bosnia and Herzegovina; violeta.soljic@fzs.sum.ba (V.Š.); marina.curlin@fzs.sum.ba (M.Ć.); 3 Department of Histology and Embryology, University Clinical Hospital Mostar, Kralja Tvrtka bb, 88000 Mostar, Bosnia and Herzegovina; 4 Faculty of Pharmacy, University of Mostar, Trg Hrvatskih Velikana 1, 88000 Mostar, Bosnia and Herzegovina; ivona.ivancic@farf.sum.ba; 5 Health Centre Široki Brijeg, Dr. Jure Grubišića 11, 88220 Široki Brijeg, Bosnia and Herzegovina; domo.tomi@gmail.com

**Keywords:** endothelial progenitor cells, coronary artery disease, acute coronary syndrome, Syntax Score, biomarker, vascular repair

## Abstract

Endothelial progenitor cells (EPCs) play a crucial role in vascular repair, and their depletion has been involved in coronary artery disease (CAD) severity. This study examines the relationship between circulating EPC levels and CAD complexity, as quantified by the Syntax Score I. A total of 85 patients undergoing coronary angiography were enrolled, with EPCs quantified using flow cytometry. EPC proportion showed a significant inverse relationship with CAD severity, measured by Syntax Score I. Additionally, we investigated EPC levels in patients presenting with acute coronary syndrome (ACS) and found that EPC depletion was more pronounced in this group compared to non-ACS patients (median EPC count: 0.35 vs. 0.61, *p* = 0.027). These findings suggest that lower EPC levels are indicative of more severe CAD and ACS, reinforcing their potential as biomarkers for cardiovascular risk stratification, monitoring disease advancement, and identifying patients at risk of adverse events.

## 1. Introduction

Coronary artery disease (CAD) remains the leading cause of global mortality, driven primarily by endothelial dysfunction and atherosclerosis. Inflammatory activation and endothelial dysfunction play key roles in CAD progression, contributing to the development of atherosclerotic plaques and increasing the risk of cardiovascular events [1]. It is characterized by the progressive narrowing of coronary arteries due to plaque formation, leading to impaired myocardial perfusion and an increased risk of adverse cardiovascular events, including myocardial infarction and heart failure [2]. Acute coronary syndrome (ACS) represents an acute manifestation of CAD, typically resulting from plaque rupture, thrombus formation, and sudden coronary occlusion. It encompasses a spectrum of clinical conditions, including unstable angina (UA), non-ST-segment elevation myocardial infarction (NSTEMI), and ST-segment elevation myocardial infarction (STEMI). The underlying mechanisms of ACS involve endothelial dysfunction, inflammation, and platelet aggregation, all of which contribute to the rapid progression of coronary thrombosis and myocardial ischemia [3,4]. Given the critical role of endothelial repair in maintaining vascular integrity, endothelial progenitor cell (EPC) depletion may contribute to worse outcomes in ACS patients, limiting their ability to recover from acute ischemic events. Endothelial dysfunction is a key driver in all stages of atherosclerosis, from early plaque formation to advanced thrombosis, highlighting its critical role in cardiovascular risk stratification [5]. The endothelium plays a crucial role in vascular homeostasis, and its repair mechanisms depend on EPCs, which originate from the bone marrow and contribute to vascular regeneration [6].

Current risk stratification for CAD relies on clinical and imaging-based parameters, yet there is a growing interest in circulating biomarkers that reflect endothelial dysfunction and vascular repair capacity. EPCs, a subset of bone-marrow-derived progenitor cells, contribute to endothelial regeneration and neovascularization, playing a crucial role in vascular repair. Their depletion has been linked to cardiovascular disease progression, highlighting their potential as biomarkers for endothelial function and cardiovascular health [7]. Several studies have suggested that a reduced number of EPCs in peripheral blood is associated with an increased risk of CAD progression. EPC depletion may reflect impaired vascular repair mechanisms, increased oxidative stress, and systemic inflammation, all of which contribute to endothelial dysfunction [8]. Emerging evidence suggests that EPC count may serve as a surrogate marker for endothelial function, with potential implications for guiding therapy. Interventions such as statin therapy, exercise, and regenerative medicine approaches (e.g., stem cell therapy) have been shown to enhance EPC levels, offering possible avenues for targeted cardiovascular risk reduction [9]. Recent findings suggest that EPC depletion is particularly relevant in ACS, where reduced EPC counts have been observed in patients with unstable plaques and recurrent ischemic events [10,11]. In this study, we aimed to investigate whether measuring EPC levels can further refine risk stratification and guide clinical management strategies in patients with coronary artery disease.

## 2. Materials and Methods

This study was designed as a prospective observational analysis conducted over a period of 2 years at University Clinical Hospital Mostar. Prior to enrollment, all participants provided written informed consent, and their data were anonymized for confidentiality purposes. Patients were considered eligible for inclusion if they were between 40 and 75 years of age and had suspected or confirmed CAD requiring coronary angiography. In total, 85 patients were enrolled in this study, of whom 59 patients (69%) presented with ACS, defined based on clinical presentation, electrocardiographic changes, and elevated cardiac biomarkers (troponin I above the 99th percentile of the upper reference limit) [12]. The remaining 26 patients (31%) comprised the control group consisting of patients with stable CAD or a normal coronary angiography (Table 1).

Stable CAD patients had evidence of obstructive coronary disease but no acute ischemic symptoms at the time of enrollment, while the control group comprised individuals without significant coronary artery stenosis as confirmed by coronary angiography. The exclusion criteria included the presence of malignancy, chronic inflammatory diseases, autoimmune disorders, active infections, or any prior history of hematological or bone marrow disorders. Patients receiving immunosuppressive therapy or cytotoxic drugs were also excluded to minimize factors that may affect EPC levels.

Coronary angiography was performed by experienced interventional cardiologists via radial or femoral artery access, with procedural standardized techniques. The severity and complexity of CAD were evaluated using the Syntax Score, a well-established angiographic tool for quantifying CAD complexity. Higher Syntax Scores indicate more severe and complex coronary lesions, often requiring advanced interventional strategies. The Syntax Score was independently assessed by an interventional cardiologist blinded to patient EPC levels and following the standardized Syntax Score Algorithm. The patients were stratified into three groups based on their Syntax Score I: low (≤22), intermediate (23–32), and high (≥33) [13,14].

**Figure 1:**
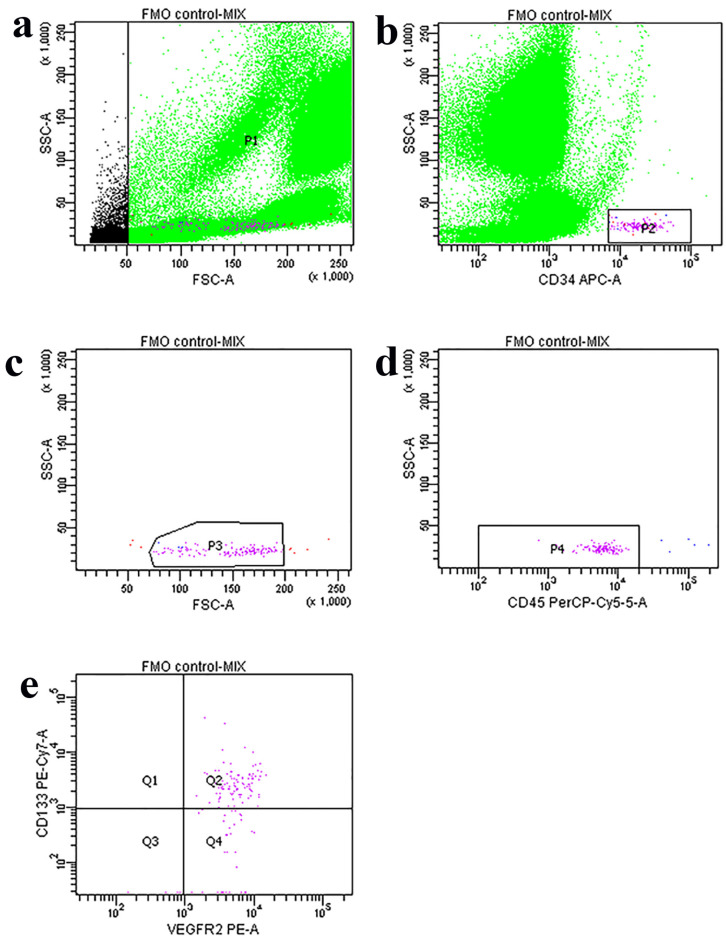
Multicolor flow cytometric analysis of endothelial progenitor cells (EPCs) in whole blood. (**a**) All leukocytes are first gated on an FSC/SSC dot plot as P1. (**b**) This P1 is displayed on an SSC/CD34 dot plot following gating of the CD34+ events as P2. (**c**) The events of P3 are subsequently presented on a FSC/SSC dot plot and gated as P3 in order to confirm the lymph-blast scatter region and to remove residual debris in front of the population. (**d**) The events of P3 are then plotted again on an SSC/CD45 dot plot and only the CD45-/dim cells are gated as P4. (**e**) These CD45-/dimCD34+ events are shown on a CD133/VEGFR2 dot plot.

Blood samples were collected from the participants immediately after coronary angiography. Peripheral venous blood was drawn into EDTA tubes and processed within 2 h of collection to ensure optimal cell viability. A 100 μL sample of whole blood was incubated in the dark at room temperature for 30 min with the following antibodies: 5 μL of PE anti-human CD309 (VEGFR2) (cat. no. 359904, BioLegend, San Diego, CA, USA), 5 μL of PerCP-Cy5.5 anti-human CD45 (cat. no. 332784, BD, Franklin Lakes, NJ, USA), 5 μL of APC anti-human CD34 (cat. no. 343608, BioLegend), and 5 μL of PE/Cy7 anti-human CD133 (cat. no. 372810, BioLegend). The sample was then treated with RBC Lysis Buffer (cat. no. 420301, BioLegend). Fluorescence Minus One (FMO) was used as a control. EPCs were quantified using flow cytometry, specifically identifying cells co-expressing as CD34+/VEGFR2+/CD133+/CD45-dim. The numbers of EPC cells were analyzed in the lymph-blast scatter region using FACS Diva software v.6.0 (BD Biosciences, Franklin Lakes, NJ, USA) and expressed as positive events/10^6^ total events (Figure 1) [15,16].

All statistical analyses were conducted using IBM SPSS Statistics v26.0 (Armonk, NY, USA). The normality of continuous variables was assessed using the Shapiro-Wilk test. Depending on the distribution, continuous variables were presented as mean ± standard deviation (SD) for normally distributed data or median with interquartile range (IQR) for non-normally distributed data. The relationship between EPC count and CAD severity was assessed using linear regression analysis. Log-transformation was performed on EPC levels due to their non-normal distribution and presence of significant outliers. To further analyze ordinal associations, Kendall’s Tau-b test was used when the Syntax Score values were categorized into three groups (low, intermediate, high). To evaluate EPC depletion in ACS, the Mann-Whitney U test was performed to compare EPC levels between ACS and non-ACS patients. A *p*-value of <0.05 was considered statistically significant for all analyses. Artificial Intelligence (AI), specifically ChatGPT-3.5 (OpenAI, San Francisco, CA, USA), was used to assist with the refinement of the language (AI did not contribute to the study design, data collection, results analysis, or formulation of conclusions).

## 3. Results

A total of 85 patients were included in the study, with a mean age of 60 ± 11 years. The cohort consisted of 77 male (91%) and 8 female (9%) participants. Baseline demographic and clinical characteristics were analyzed across the different Syntax Score groups. No significant differences were observed in baseline characteristics among the groups, suggesting comparable patient profiles in terms of traditional cardiovascular risk factors.

### EPC Proportion and Syntax Score Correlation

The EPC proportion in peripheral blood changes depending on coronary artery disease severity and clinical presentation (Table 2).

In the linear regression analysis, the EPC proportion in peripheral blood showed a significant inverse relationship with CAD severity, measured by Syntax Scores. A one-unit increase in log (EPC) is associated with a 3.54-point-associated decrease in Syntax Score I (R^2^ = 0.056, *p* = 0.029) (Table 3).

**Figure 2:**
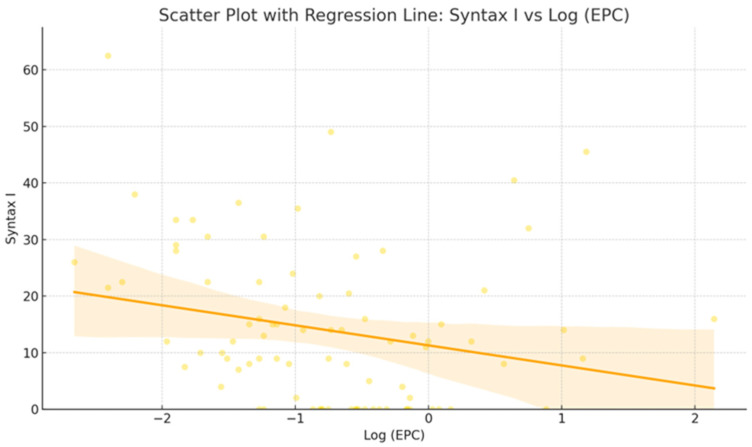
Scatter plot with regression line showing the inverse relationship between EPC proportion and degree of coronary artery disease (CAD).

These findings suggest that lower EPC levels are linked to greater CAD complexity and severity (Figure 2).

**Figure 3:**
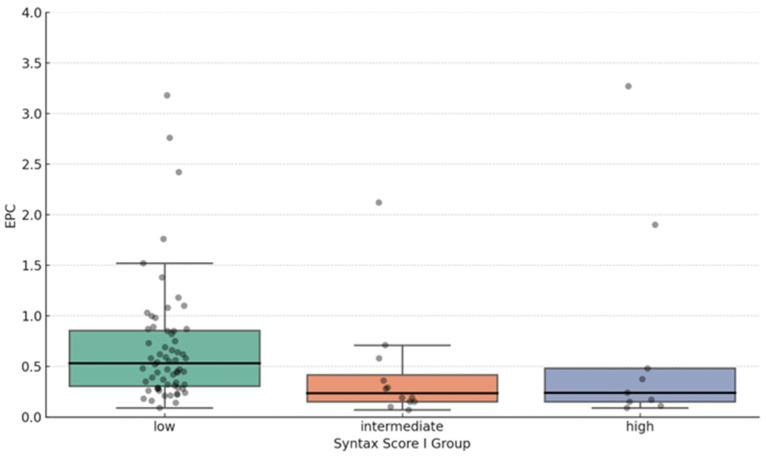
Correlation between EPC share and Syntax Score I scale categories. The low Syntax score group has a higher median EPC proportion (0.54) than the intermediate (0.23) and high-risk group (0.24).

In further analysis, the values from the Syntax Score I were categorized into three groups. Syntax Score I values less than 22 were categorized as low Syntax values; those from 22 to 32 were intermediate; and those more than 32 were high Syntax values (Figure 3). Using the Kendall’s Tau-b test, we observed a statistically significant negative correlation between EPC proportions and Syntax Score I (Kendall’s Tau-b = −0.250, *p* = 0.004).

**Figure 4:**
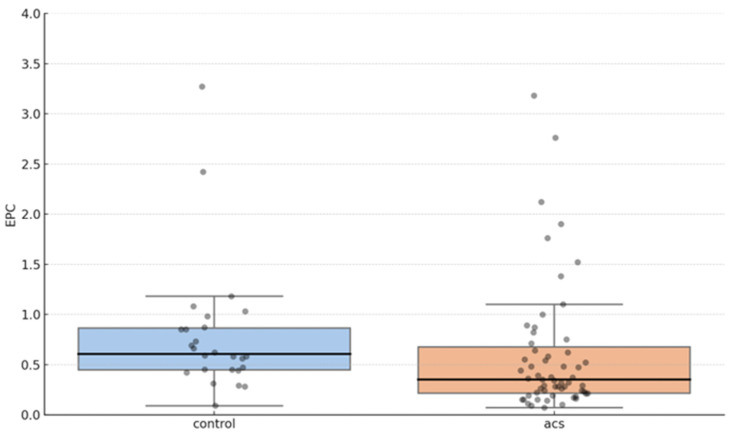
Differences in the proportion of EPCs in patients with acute coronary syndrome (ACS) and the control group. Median (IQR) for the control and ACS group are 0.61 (0.45–0.87) and 0.35 (0.22–0.68), respectively.

In addition to CAD severity, we analyzed EPC levels in ACS patients. The Mann–Whitney U test revealed that the EPC counts were significantly lower in ACS patients compared to those with stable CAD or normal angiography (median EPC count: 0.35 vs. 0.61, *p* = 0.027). A graphical representation (Figure 4) further illustrates this difference, demonstrating a distinct shift toward lower EPC proportions in the ACS group.

## 4. Discussion

This study provides evidence that EPC counts are significantly reduced in patients with more severe CAD, as assessed by the Syntax Score I. The observed negative correlation between EPC count and Syntax Score I suggests that patients with advanced atherosclerosis exhibit impaired vascular repair mechanisms, exacerbating endothelial dysfunction.

EPCs contribute to endothelial regeneration, and their depletion has been linked to increased cardiovascular risk [7,17]. Our findings align with those of previous research indicating that lower EPC levels correlate with atherosclerotic disease severity and are inversely associated with endothelial function [18,19]. This trend suggests that EPC depletion may reflect worsening endothelial dysfunction, oxidative stress, and systemic inflammation, all of which contribute to CAD pathogenesis [4,5].

The Syntax Score, an established tool for assessing CAD complexity, correlates with EPC depletion, implying a reduced capacity for endothelial repair in patients with high Syntax Scores [14]. While this association highlights a potential prognostic value of EPC counts, further studies are needed to determine their predictive power for adverse cardiovascular events.

In addition to CAD severity, EPC depletion was significantly more pronounced in patients with ACS compared to those with stable CAD (median EPC count: 0.35 vs. 0.61, *p* = 0.027). These results suggest that EPC depletion may be indicative of heightened plaque instability, endothelial dysfunction, and increased thrombotic risk in ACS patients [10,11]. Prior studies have shown that lower EPC levels are linked to poorer post-ischemic angiogenesis and worse clinical outcomes [20], reinforcing their potential as biomarkers for cardiovascular risk assessment.

Several studies, including those by Vasa et al. [17] and Hill et al. [18], have demonstrated that EPC depletion is associated with traditional cardiovascular risk factors such as hypertension, hyperlipidemia, smoking, and diabetes. Furthermore, therapeutic interventions such as statins, exercise, and lifestyle modifications have been shown to enhance EPC levels, suggesting a role for EPC monitoring in guiding treatment strategies [21,22].

Given these findings, EPC quantification may complement existing CAD and ACS risk stratification models, potentially identifying high-risk patients who would benefit from aggressive preventive strategies [23,24]. Given our findings, EPC levels may serve as a supplementary biomarker in CAD risk stratification, potentially guiding early interventions to prevent disease progression. Future research should focus on longitudinal studies assessing whether EPC depletion predicts long-term cardiovascular outcomes, as well as interventional trials exploring EPC-targeted therapies such as regenerative medicine and endothelial repair strategies [25].

However, some studies have reported conflicting findings regarding the relationship between EPC levels and CAD severity. Fadini et al. [23] found no significant correlation between EPC counts and CAD severity when adjusting for traditional cardiovascular risk factors, suggesting that other mechanisms may play a role in disease progression. Additionally, Xiao and Kuang [26] argued that EPC functionality (e.g., migration, adhesion) may be more relevant than absolute EPC count in vascular repair. These differences could be due to variations in study populations, EPC identification techniques, or the relative impact of other cardiovascular risk factors.

While our study provides valuable insights, it has some limitations. The relatively small sample size may affect generalizability, and variability in EPC quantification methods could introduce measurement bias [15]. Additionally, while we adjusted for key cardiovascular risk factors, other unmeasured variables such as inflammatory markers and lifestyle habits may also influence EPC levels [5].

## 5. Conclusions

This study demonstrated that EPC depletion is significantly associated with the severity of CAD, as assessed by Syntax Score I. The observed inverse correlation for Syntax Score I suggests that lower EPC levels may reflect impaired vascular repair mechanisms, increased oxidative stress, and systemic inflammation in patients with more advanced coronary atherosclerosis. Furthermore, our findings indicate that EPC depletion is more pronounced in ACS patients, with significantly lower EPC counts compared to those with stable CAD. This suggests a potential link between EPC depletion, plaque instability, and increased thrombotic risk in ACS, reinforcing their potential role as biomarkers for cardiovascular risk assessment. While the results support the use of EPC quantification as an addition in CAD and ACS risk stratification, further research is needed to evaluate its clinical utility in predicting adverse cardiovascular outcomes and guiding therapeutic interventions. Larger, longitudinal studies are required to validate these findings and explore the impact of EPC-targeted therapies in improving patient outcomes.

## Figures and Tables

**Table 1:** Clinical features of patients in study group.

	N = 85 Patients
Sex	Male 77 (91%) Female 8 (9%)
Smokers	34 (40%)
Acute coronary syndrome	59 (69%)
STEMI NSTEMI UA Control group	38 (45%) 13 (15%) 8 (9%) 26 (31%)
Age (yr)	60 ± 10.7
Height (cm)	180.3 ± 8.3
Weight (kg)	92.7 ± 14.3
BMI (kg/m^2^) Acetylsalicylic acid Clopidogrel Statins	28.5 ± 4 63 (74%) 59 (69%) 67 (79%)
Systolic blood pressure (mmHg)	128.1 ± 18
Diastolic blood pressure (mmHg)	78.1 ± 8.7

yr—years; kg—kilograms; cm—centimeters; BMI—body mass index; mmHg—millimeters of mercury. Data presented as mean ± SD.

**Table 2:** Endothelial progenitor cell proportion in peripheral blood (10^6^ cells analyzed per sample).

Proportion of EPC × 10^−2^—median (IQR)	
All patients (N = 85)	0.45 (0.26–0.82)
Syntax Score I groups	
Low	0.53 (0.31–0.86)
Intermediate	0.23 (0.15–0.42)
High	0.24 (0.15–0.48)
Coronary artery disease groups	
ACS (N = 59)	0.35 (0.22–0.68)
Control group (N = 26)	0.61 (0.45–0.87)

**Table 3:** Linear regression analysis of the relationship between EPC proportion in peripheral blood and Syntax Score I (R^2^ = 0.056, *p* = 0.029).

Variable	Coefficient	Std. Error	t-Statistic	*p*-Value
Intercept	11.28	1.87	6.03	<0.001
Log (EPC)	−3.54	1.59	−2.22	0.029

## Data Availability

The data supporting the findings of this study are available from the first author upon reasonable request. The data will be available for five years after publication.
